# Inadequate programming, insufficient communication and non-compliance with the basic principles of maternal death audits in health districts in Burkina Faso: a qualitative study

**DOI:** 10.1186/s12978-017-0379-1

**Published:** 2017-09-29

**Authors:** Boukaré Congo, Djénéba Sanon, Tieba Millogo, Charlemagne Marie Ouedraogo, Wambi Maurice E. Yaméogo, Ziemlé Clement Meda, Seni Kouanda

**Affiliations:** 1 Institut Africain de Santé Publique (IASP), Ouagadougou, BP 199 Burkina Faso; 2 Ministère de la santé, Ouagadougou, Burkina Faso; 3 Institut de Recherche en Sciences de la Santé, Ouagadougou, 03 BP 7192 Burkina Faso; 4 UFR/SDS, Université de Ouagadougou, Ouagadougou, BP 7021 Burkina Faso; 5 INSSA, Université Polytechnique de Bobo-Dioulasso, Bobo-Dioulasso, Burkina Faso

**Keywords:** Audit, Maternal deaths, Burkina Faso

## Abstract

**Background:**

Implementation of quality maternal death audits requires good programming, good communication and compliance with core principles. Studies on compliance with core principles in the conduct of maternal death audits (MDAs) exist but were conducted in urban areas, at the 2nd or 3rd level of the healthcare system, in experimental situations, or in a context of skills-building projects or technical platforms with an emphasis on the review of “near miss”. This study aims to fill the gap of evidence on the implementation of MDAs in rural settings, at the first level of care and in the routine care situation in Burkina Faso.

**Methods:**

We conducted a multiple-case study, with seven cases (health districts) chosen by contrasted purposive sampling using four criteria: (i) the intra-hospital maternal mortality rates for 2013, (ii) rural versus urban location, (iii) proofs of regular conduct of maternal death audits (MDAs) as per routine health information system, and (iv) the use of district hospital versus regional hospital for reference when the first mentioned does not exist. A review of audit records and structured and semi-structured interviews with staff involved in MDAs were conducted. The survey was conducted from 27 April to 30 May of 2015.

**Results:**

The results showed that maternal death audits (MDAs) were irregularly scheduled, mostly driven by critical events. Overall, preparing sessions, communication and the conduct of MDAs were most of the time inadequate. Confidentiality was globally respected during the clinical audit sessions. The principle of “no name, no shame, and no blame” was differently applied and anonymity was rarely preserved.

**Conclusion:**

Programming, communication, and compliance with the basic principles in the conduct of maternal death audits were inadequate as compared to the national standards. Identifying determinants of such shortcomings may help guide interventions to improve the quality of clinical audits.

**Resume:**

La mise en œuvre d’audits de décès maternels de qualité nécessite une bonne programmation, une bonne communication et le respect des principes fondamentaux. Des études sur le respect des principes fondamentaux existent mais ont été menées dans les zones urbaines, le 2ème ou 3ème niveau du système de santé, dans des situations expérimentales, un contexte de projets de renforcement des compétences ou de plates-formes techniques, en mettant l’accent sur la revue des «near miss». Cette étude vise à combler le manque d’information sur la programmation et le respect des principes fondamentaux concernant le milieu rural, le niveau du système de santé qui est. le district sanitaire et la situation de routine au Burkina Faso.

**Méthodologie:**

Nous avons mené une étude de cas multiple dans 7 établissements de santé sélectionnés par échantillonnage raisonné contrasté selon 4 critères: milieu urbain ou rural, taux de mortalité maternelle dans les établissements de santé en 2013 (les données de l’année 2014 n’étant pas complètes à la rédaction du protocole), la déclaration des audits de décès maternels dans le système de surveillance nationale, le recours ou non par le district choisi à un centre hospitalier régional pour les soins complémentaires de premier niveau (normalement offerts à l’hôpital de district s’il existe). Une revue des dossiers d’audits, ainsi que des entretiens directifs, semi-directifs auprès du personnel impliqué dans les soins de maternité ont été réalisés. L’enquête s’est. déroulée du 27 Avril au 30 Mai 2015.

**Résultats:**

Les résultats montrent que les revues des décès maternels ont été irrégulièrement programmées, de façon espacée et très souvent au gré des évènements. La préparation, la conduite des séances et la communication après les séances ont été défaillantes. La confidentialité au sein du groupe d’auditeurs a été respectée tandis que le niveau de respect du principe de « no name, no shame, no blame » a varié d’une structure à une autre. Enfin, l’anonymat a été le moins respecté.

**Conclusion:**

La programmation, la communication et le respect des principes fondamentaux ont connu des défaillances par rapport aux normes mais de façon variable d’une structure à une autre. L’identification des déterminants de ces insuffisances pourront aider à l’orientation des interventions visant l’amélioration de l’activité des audits de décès maternels au niveau district de santé.

## Plain summary

The implementation of good quality maternal death audits (MDAs) requires good programming, good communication and adherence to core principles. Studies were conducted on the compliance with core principles in the implementation of MDAs in urban areas, at the second or third levels of the health care system or in experimental context. Our study sought to fill the gap of knowledge on the level of adherence to core principles in the implementation of MDAs in rural areas, at the peripheral level (health district) and in routine care situation in Burkina Faso.

We conducted a qualitative multiple-case study, with seven cases (health districts) chosen by contrasted purposive sampling using four criteria: (i) the intra-hospital maternal mortality rates for 2013, (ii) rural versus urban location, (iii) proofs of conduct of maternal death audits (MDAs) as per routine health information system, and (iv) the use of district hospital versus regional hospital for reference when the first mentioned does not exist. A review of audit records and in-depth individual interviews with staff involved in maternity care were conducted.

We found that the MDAs sessions were irregularly scheduled and tend to be organized only when favourable circumstances are present such as funding by a partner. Preparatory sessions for MDAs and communication around the activity were inadequate as compared to relevant standards. Confidentiality was overall respected. The principle of “no name, no shame, and no blame”, was differently applied in the health facilities. Finally, anonymity was the core principle that was lesser adhered to. Our study showed the need to improve the quality of maternal death audits in improving adherence to standards in order to achieve a significant reduction in maternal deaths.

## Background

Maternal mortality rates remain very high globally and particularly in sub-Saharan Africa [1]. Most of these deaths are avoidable [2, 3]. The combat against maternal mortality involves several strategies including implementation of audits of maternal death aiming to improve the quality of care [3–5]. The quality of these maternal death audits (MDAs) requires adherence to core principles (confidentiality, anonymity, non-accusation and lack of punishment), rules for communication between health workers and a good scheduling of audits sessions [3–6]. Several Studies have investigated the adherence to core principles in the conduct of MDAs in Tanzania [7], South Africa [6], Nigeria [8], Benin [9], and Burkina Faso [10]. All these studies were conducted in urban areas, at the 2nd or 3rd level of the healthcare system, or in experimental situations and/or in a context of skills-building projects or technical platforms with an emphasis on the review of “near miss” [9–11]. This research aims to assess the quality of the programming, the communication, and the compliance with the basic principles of MDAs according to the national standards for health districts, the 1st level of the healthcare system, in Burkina Faso.

## Methods

### Programming and basic principles of audits

In the national reference documents related to clinical audits (including the review of cases of maternal deaths in health facilities), the following points stand for standards: confidentiality of information during the clinical audits, anonymity (“No name”) of the cases audited by the audit committee members, no accusation (“no shame”), and no punishment (“no blame”). Principles and rules of good conduct of clinical audit (mutual respect, openness, active participation, acceptance of discussion and questioning of practices ...) have been specified in an “Audit Charter” whose reading and approval is required prior to participating in any audit sessions. Audits or reviews are meant to be regularly scheduled with an interval of one to three months. The process should involve the maximum of stakeholders involved with the case management and/or by the decision making in order to facilitate the implementation of the recommendations. Communication between the different actors of maternal health before and after audits is also important. Feedback of maternal audits to stakeholders for finding solutions to the problem should be carried out and has been advocated for by the Direction of Family Health of the Ministry of Health. An outline for good conduct of the session of MDAs was established. It emphasizes the need for an environment ensuring confidentiality, role sharing, good management of discussions in compliance with the principles and rules stated in the charter [3–5].

### Study setting

The study was conducted in Burkina Faso, a landlocked developing country in West Africa. Its public health system includes an administrative and operational organization.

Administratively we have a central level (constituted by the minister’s office, the general secretariat and the central directorates), an intermediate level composed of the Regional Directorates of Health and the Regional Hospitals, and a peripheral level with 70 health districts, 63 of which are headed by district teams.

Healthcare provision is equally organized in three levels. The first level is composed of two echelons (the first echelon with 1643 primary health care facilities and the second echelon with 47 Medical Centers with Surgical Antenna (MCSA) or District Hospitals). The first and the second echelons are under the responsibility of the health district. Some health districts do not have district hospital as referral center and would refer patients requiring additional care directly to the corresponding regional hospital that stands for their district hospital. A total of nine health districts are in the latter described situation, referring directly to the regional hospital, which pertains in the health care system to the second level. A total of nine functioning regional hospitals are in the country. The third level of care consists of one national hospital and three university teaching hospitals.

In the context of health district, maternal deaths may occur in the community, in primary health care facilities, in MCSU or in Regional hospitals when the latter stand for direct referral centre for the health district. To date, the country has 63 functioning health districts headed by health district management teams [12–14].

### Study design

We carried out a cross sectional qualitative multiple cases study from 27 April to 30 May of 2015. The cases are health districts purposely chosen between those that reported carrying out MDAs. Cases selection was carried out in a way to have a mix of rural and urban location health districts, districts with high, average and low intra-hospital maternal mortality rates and districts using district hospitals and regional hospitals for reference.

### Study populations

We analyze all maternal deaths that occurred from January 1st to December 31st 2014 and we selected a sample of the staff members involved with healthcare provision to women in the maternity wards in all type of health facilities in the selected health districts.

### Sampling

#### Selection of study sites

The health districts were selected using contrasted purposive sampling based on the declarations of the MDAs performed in 2013, the intra-hospital maternal mortality rates for the year 2013, and the location area of the health district (urban vs rural). Five groups of health districts were constructed based on the reported intra-hospital maternal mortality rates (close to the minimum national rate, close to the national average rate and close to the maximum national rate) and the location area. The regional hospitals were considered also if the selected health districts did not have a MCSU as referral center. We selected 5 health districts with district hospitals as referral center (Djibo, Tougan, Dafra, Tenkodogo, Ouahigouya) and 2 health districts that use regional hospitals as referral centers (Tenkodogo and Ouahigouya) to be included in the study.

#### Selection of maternal deaths

In the selected districts, all maternal deaths that occurred in healthcare facilities in 2014 and for which a review was carried out were included in the study. Reviews conducted should have a minimum of proof documents (charter, attendance list, clinical case summary, case analysis or discussion summary sheet, micro-planning for implementing solutions, change evaluation sheet) available regardless their completeness or nature (soft and/or hard copies). Documents (memos, posters, activity reports, etc.) in paper or electronic form related to maternal deaths and/or audits were also reviewed.

#### Selection of respondents

Participants were selected among the staff involved in the maternity unit (delivery room, chirurgical ward, and postoperative care units), staff of pharmacy, staff of laboratory, and staff from administration and management sections at the MCSU or regional hospitals levels in each selected health district. The heads of the previous mentioned departments were systematically included in the study. When an audit committee was in place and functioning, all the members of such committee were included in the study. Additional respondent selection in each unit was conducted in a way to represent all available qualifications in the health facility. The number of respondents to be included in the study and their respective qualifications for each health district was informed by a quick review of the general information obtained on human resources and the organization in place for the conduct of MDAs.

### Data collection techniques and tools

the data collection took place from 27 April to 30 May of 2015. General information on health facilities were obtained through face-to-face interviews with key respondents at each health facility level using a questionnaire.

An interview guide (see Table 1) was used to assess the compliance with known standards in the conduct of MDAs using in-depth individual interviews (IDIs). The IDIs were conducted by a medical doctor who was a public health student at the time of the study and was previously trained on MDAs. Full information on the themes and sub-themes that were investigated during the IDIs and the targets for each question are available in Table 1. In addition to the previous mentioned techniques, data extraction from relevant data sources (patients ‘charts, audits records etc.) was performed to complete the information gathered through IDIs. The interviews were conducted in French and tape recorded using a Dictaphone.

**Table 1:** Interview guide

Questions	Possible probes	Targets
1. Identity and other general information related to maternal death audits.	- Qualification - Service, - Technical post, - Responsibility, - Duration in the: profession, service, technical post, position of responsibility, - Was trained on MDAs, - Year and place of training on audits - Experience in the practice of audits,	- All interviewees
2. What is your definition (s) for MDAs and what are their interests?	- Definition of audits - Interest of audits	- All interviewees
3. How do you perceive the conditions under which audits are carried out in your structure?	- Human resources (number, training, knowledge, involvement, motivation) - Logistic and financial resources - Coordination and monitoring of activities - Importance of maternal mortality	- All interviewees
4. How are audit sessions prepared in your structure?	- How information on conducting audit meetings is given? - How are cases identified? - How are informations about the case collected? - How participants in the audit session are identified?	- Head of health districts/regional hospitals - Member of the Audit Committees, - Heads of MCSUs, - Heads of maternity units.
5. How are audit sessions organized in your structures?	- How is the choice of where audit session has to be hold? - How is the audit site prepared? - How are the roles distributed during the audit session? - How is speech management done during the audit? - How do you like the atmosphere in which the audit sessions take place?	- Persons who have already participated in a maternal death audit.
6. How are maternal deaths examined in practice during the audit session (steps of the audit cycle)?	- What are your references for these audits? (Standards and reference protocols) - How are the data on the case audited (clinical summary) delivered? - How is the case analyzed? (Dysfunctions, causes, solutions) - How do you solve problems? (Resolution plan, restitution, monitoring of implementation, assessment of changes)	- Persons who have already participated in a maternal death audit.
7. How are agents and teams (that have been involved in the treatment of women who have died and who have been audited) treated before, during and after the auditing sessions?	- How do you appreciate the respect of anonymity before, during and after the audit sessions in your district, your health structure? - How do you appreciate the lack of shame before, during and after the audit sessions in your district, your health facility? - How do you appreciate the lack of blame before, during and after the audit sessions in your district, your health facility?	- All interviewees
8. What conclusion do you make about the practice of auditing maternal deaths in the district?	- What are the positive aspects and their causes? - What are the negative aspects and their causes? - What are your suggestions / recommendations?	- All interviewees

### Data processing and analysis

The data recorded were transcribed and enter into MS Word. The data were thereafter render anonymous using codes. Health facilities were numbered from 1 to 7. The number of health facility followed by and order number was used for interviewees. We performed a framework analysis in accordance with the four dimensions of the principles of maternal death audits: general conditions of conduct of audits, principles of audits, stages of the audit cycle, overall appraisal of the practice of audits. Each dimension was declined into subdimensions (Table 2). Following these dimensions and subdimensions, we developed an analysis frame with MS Excel 2010 to code the data. To do this, an analysis frame (based on the basics principles of maternal death audit was developed, theme by theme.

**Table 2:** Themes, sub-themes and levels used in the analysis of data

Themes	Subtopics	Levels
General conditions of conduct of audits	Human resources	Knowledge of audits Numbers Qualifications Continuing education / supervision Workload, Involvement in audits Motivation
	Appraisal of the materials	
	Assessment of financial resources	
	Coordination and monitoring of activities	
	Extent of maternal mortality	
Principles of audits	Confidentiality	
	Anonymity	
	No discrimination	
	Non-stigma	
Preparation and organization of audit meetings	Communication on audit meetings	Communication before the meetings Communication during the sessions Post-session communication
	Identification of participants in the session	
	Identification of cases to be audited	
	Room (location selected for audit)	
	General working atmosphere during the session	
Stages of the audit cycle	Collection of data on the case to be audited	Process of collection, Clinical Summary
	Data analysis	Identification of malfunctions Identification of causes of malfunction, Identification of solutions, Synthesis of the analysis, Conclusion on the causes and factors that contributed to the death, Conclusion on the avoidability or otherwise of death, Recommendations
	Dissemination and Implementation Plan	
	Assessment of implementation and results	
Overall appraisal of the practice of audits		
Suggestions / recommendations		

### Ethical considerations

The National Ethics Committee for Health Research in Burkina Faso in its statement n° 2015-5-058 authorized this study. The interviews were only conducted after obtaining an informed and written consent. During the data collection and analysis the anonymity and confidentiality of study participants were safeguarded and all informations are stored on laptops protected by passwords. Access and utilization of the data collected was limited to the research team.

## Results

### Characteristics of the study sample

The interviews were conducted on a total of 73 participants. The Table 3 shows the interviewee’s characteristics. The Seven health facilities recorded a total of 145 maternal deaths from January to December 31 of the year 2014 of which 31.72% (46 deaths) were reviewed.

**Table 3:** Background characteristics of the study sample

Characteristics		Health districts							Total
		^a^1	2	3	4	5	6	7	
Total number of interviewees		10	5	15	17	3	10	13	73
Persons with responsability post		6	5	7	6	3	5	5	37
Audit committee members		No committee	No committee	6	5	No committee	4	No committee	15
Females		4	1	4	5	0	3	7	24
working position	Healthcare providers	6	3	10	10	1	6	8	54
	OtherStaffs	4	2	5	7	2	4	5	29
Duration in the Position the job	< 1an	1	1	0	1	0	1	1	5
	≥ 1an	9	4	15	16	3	9	12	65
Persons trained on audits		4	0	7	11	3	0	1	26
Period of training	≤ 2014	4	0	7	2	3	0	1	17
	2015	0	0	0	9	0	0	0	9
Persons who have participated at audits in 2014		2	2	5	2	0	4	4	19
Total of MDAs in 2014		29	3	27	61	3	13	9	145
Total of MDAs in 2015		9	2	3	13	0	11	8	46

^a^Case or healthcare facilities are numbered from 1 to 7

### Audit programming

Varying timings were used for the programming of MDAs’ sessions across health districts (HD). Clinical audit sessions were held twice a year in four health districts (1, 2, 3, and 4). One facility (7) conducted only one session in 2014. In another one (6), reviews were regularly organized within one-month time following the occurrence of the maternal death. All type of audits (death and near miss reviews) were pooled together in the form of seminars in all facilities except one health facility (6) maternal deaths and near miss are reviewed separately.

### Communication before audit sessions

In all the health Facilities, « an invitation to participate in MDAs sessions was always issued to all stakeholders » according to 6/7 heads of health districts interviewed. The only exception to that was noted in HD 5 that reported the conduct of MDAs in the routine health information system but could not show any evidence of MDAs conducted in 2014. The qualifications of participants invited to participate in MDAs sessions varied from one facility to another. In HD 3 and 6, all health care providers working in the maternity, surgical, pharmacy, laboratory and administrative units and willing to participate were invited. In HD 1 and 3, only a number of selected persons termed as “qualified for MDAs” were invited to participate in MDAs. In HD 7, heads of healthcare units and a sample of healthcare providers working in the maternity wards were concerned with the MDAs.

Medical doctors, the head of the department of reproductive health and all the heads of units of care where a case of maternal death occurred are invited to participate in the MDAs sessions in HD 2.

The information channels used are not always performing well. That was acknowledged by an official as quoted below:

“We used to share the information on the sessions using mobile phones and staff meetings. However, many were complaining that they don’t get the information. Now we used information notes that are posted in each unit concerned with the review” (A medical doctor responsible for audit of maternal deaths in a MCSU).

The restriction of participation in MDAs was identified by some officials and health care providers as a shortcoming. A Health care provider in one regional hospital said: “we used to involve health care providers working in the maternity units, since they are not trained, the contribution was few. We decided to conduct now the audits in small teams, the other health care workers are unhappy about that, and they are right”.

Some actors don’t feel concerned with the audits because they have not been formally invited to participate or because of misconceptions regarding MDAs. For one interviewee: « we are not concerned with the reviews; it’s an activity that concerns the maternity ». For another actor, MDA « is an activity of those involve in clinical care ». For this actor working at the pharmacy unit: « we don’t see the importance of pharmacy in this activity » “we think the management section has nothing to do with audits”.

### Communication during audit sessions

A framework for facilitating sessions is suggested in the national auditing guide. Use of the charter, the establishment of the attendance list, and summary of the discussions about the case were assessed through reviews’ documents. The charter that is expected to guide the participants’ behaviour during and after sessions was found in only 5 HD (1, 3, 4, 6, and 7), and properly used in only one of them (7) where it was read and approved with the signature of all participants. Attendance lists were not found in 3 HD (1, 2, and 3). The Table 4 showed the summary of the availability and use of the audit charter in HD.

**Table 4:** Use of the audit charters in health facilities

Characteristics		Health Districts					
		^a^1	2	3	4	6	7
Hardcopy of the charter found		No	No	No	No	Yes	Yes
Electronic version of the charter found		Yes	No	Yes	Yes	No	Yes
Hardcopy version approved with signature in the audit files		No	No	No	No	No	Yes
Minimal content found	Definition, interest and rules of conduct	Yes	^b^NA	Yes	NA	Yes	Yes
	Approval, signature of the Charter	NA	NA	NA	NA	No	Yes
Modality of practical use of the charter		Reading and oral approval	Explanation of the principles	Reading and oral approval	Reading and oral approval	Reading and oral approval	Reading and approval with signature
^c^Compliance with all the standards of use of the Charter		No	No	No	No	Non	Yes

^a^Case or healthcare facility, ^b^Non-applicable ^c^Compliance with all the standards of use of the Charter

An analysis was carried out for all audited cases. Roles (chair of session, person in charge of the report, responsible for presenting the case*,* etc.) are shared in all health districts according to the actors. No incident was noted in discussions. A physician in a rural health district argued that *“Debates were sometimes stormy but people often come to understand each other. There’s never been any unfortunate incident here at the moment”*.

Concerning participation in discussions during sessions, we constantly heard: *“participation cannot be the same for all cases and for all participants*. *Some people are often too talkative and some don’t speak at all”*. (Midwife, care unit supervisor in a regional hospital).

### Communication after audit sessions

Communication after audit sessions was focused on the presentation of findings and implementation of the recommendations. Presentation of findings was made in all HD but in different ways. The profile of the actors involved depended on the location and the circumstances around the organization of such presentations. Some facilities incorporated them into statutory meetings like: meeting of district management team and nurses heads of primary health facilities, meeting of the management team and managers of maternities, meeting of district management team, meeting of nurses heads of primary health facilities and care unit supervisors or technical unit supervisors. For the HD 1, where no financial support was available, the restitution on a large-scale of audit outcomes was conducted at the Heath Regional Direction. The number and profile of participants to this restitution depended on the location and the organization framework.

### Compliance with core principles of audits

#### Adherence to confidentiality

According to the results of the interviews, although the charter is not properly used, confidentiality of information from audits was ensured. A laboratory technician said: *“I have never heard since we’ve started audits, that an officer went and said things outside. I think we are all health workers, subject to the confidential medical information, so the problem does not arise.”* (Biomedical technologist, in a district hospital).

Compliance with this principle of confidentiality was sometimes even overstated or misunderstood. A midwife said: *“They (members of audit committee) did not even give us the results of audits. They say it is confidential and nothing must leak from the team and only the department head can talk about that.”* (Midwife, regional hospital).

#### Compliance with “no name, no shame, and no blame” principle

To assess the respect of the “no name” principle (anonymity), we analysed the audit records, the reports of audits and other activities linked to the audit sessions. This analysis showed shortcomings concerning the respect of the anonymity in two health districts (4 and 6). This was reflected on the one hand by the clear *identification of the deceased* women and citation of the clinical staff involved in audit documents. On the other hand, the anonymity was not sometimes respected as *it was possible to deduce the identity of the teams by how summaries were presented and through attendance lists in audit sessions*. For example, in the clinical summary of a case, it is stated that the only male skilled birth attendant in the zone “Z” was called for the management of the case. On the attendance list, we found “Mr. X Y”, male skilled birth attendant in the zone “Z”. Thus, we recognized that “Mr. XY” is identified for participating in the treatment of the case.

But in addition to documents reviews, respect of anonymity, discrimination and stigma was assessed through analysis of the statements of the actors. Four situations were identified.

Firstly, anonymity was properly respected in some HD. There was no discrimination or stigma (7, 3, and 2). A health care provider said: *“Here, whenever we present cases, we avoid putting the name of the patient and of the team. We avoid blaming or shaming or yelling at people”.* (Specialized nurse in surgery, on duty for 12 years, care unity supervisor).

Secondly, anonymity was not respected but the principle “no shame and no blame” was mostly adhered to (4). A midwife summarized it this way: *“Sometimes, we already know the person. Even if nobody says it, we already know it is such person, but we did not see proven cases of stigma”* (midwife, regional hospital).

Thirdly, anonymity and no shame principles were not respected but no blame was caused. A skilled birth attendant said:

*“Concerning the fundamental principles, at our level, it is not easy particularly since the medical records are often sent to the audit place and others ask to see it. So, there is no anonymity. Before finishing the meeting, you know the health worker who treated the woman. It also happens that all information doesn’t appear in the records; the team that provided care to the woman is obliged to provide clarification. At the end, the hidden side becomes useless given that you may have to add some lacking information that does not appear in the medical record such as a testimony of the woman’s relatives… Self-shaming exists, but there is no blame.”* (State maieutician, 6 years at his post in a health district, Unit Manager).

Fourthly, no name, no shame, and no blame principles were not respected. Blaming was in the form of *“calls*” (phone call or meeting of offenders with a manager, clarifications on the misconduct, warning) for workers who committed misconducts in some facilities. A doctor, in a managerial position in a health district said:

*“There is no blame as such but we question (very often, such questioning is made in the sense to ensure that the same does not happen again)”.*

Overall, confidentiality was respected in all facilities but the other principles were respected at varying degrees.

## Discussion

The results of this study showed the level of compliance with core principles and limitations in the implementation of MDAs in the context of first level of health system in Burkina Faso, West Africa.

### Audit programming

The programming of MDAs sessions was either uneven or absent in the majority of HD. Similar result on irregular conduct of MDAs was reported by Hamersveld et al. in Tanzania in 2012 [15]. The reviews were most of the time organized in the form of seminars, during which several cases of maternal deaths that occurred during a specific period are reviewed all together. This is contrary to the recommended procedures whereby MDAs audits are recommended on a regular basis and ideally within one-month time after the occurrence of the case [3–5]. Practices closer to standards in programming audit sessions were reported by Borchert et al. in Benin working on “near miss reviews”, by Hofman in Nigeria and Richard in Burkina Faso in situations of experimentation or specific interventions. The challenges encountered with the compliance to standards in programming MDAs in our study may reflect the real world situation where the lack of financial resources and logistical constraints prevail more than experimental situation [8, 11, 16]. The current approach whereby the organisation of MDAs’ sessions depends on the availability of donors funding is less likely to issue the expected outcomes. The most reasonable way would be considering MDAs as a routine and quality improvement strategy. This approach would lead to a more systematic organization of MDAs as cases occurred and their inclusion in the ordinary package of the set activities that health care providers are entitled to. Indeed, the scheduling of reviews sessions beyond the ordinary working hours is one of the reasons that justify the need to afford financial compensations for committee members as reported by Hutchinson et al. in Benin in 2010 [9].

### Communication before, during and after audit sessions

Broadening participation to include all relevant participants in MDAs is recommended in the standards [3–6] but was not always adhered to in our study. The profile of the actors invited to participate in audit sessions varied from one HD to another. A large number of stakeholders were involved in some HD while other tend to limit the participation to a restricted number of participants mainly those involved the provision of care to child birthing women in the maternity units. Several explanations were found in the support of the limitation of the number and profiles of participants among which the limited number of qualified persons (those that have been trained in MDAs), financial resources constraints and the difficulty of maintaining the confidentiality of the process if a large number of participants are involved.

A number of good interpersonal communication practices documented in the charter must be adhered to during the review [4]. The working environment and the management of the discussions must also be done following well recognized good practices [4]. The use of the charter in our study was inadequate. Richard et al. in their experimental study in Ouagadougou [14] reported inadequate interpersonal communication during the audit sessions.

Meetings to share results are hold but do not bring together all the relevant stakeholders.

Organization of sessions to disseminate findings was reported in all HD. However, the methods used for the dissemination of the results varied across HD in terms of location, settings, and participants in the meeting. The dissemination of MDAs findings was recommended by reference documents [5] and some studies [6, 17]. Its practice is however not codified [4, 5]. This may explain the differences observed between HD in our context. Therefore, Contrary to what our study found to be a common practice, audit results should be presented to as many relevant actors as possible, so that they can contribute to searching for solutions and overseeing their implementation [4, 5, 7, 17]. Some audit committees’ members were reluctant to share the results of the audits because they thought this would bring a breach into the confidentiality. This suggests there is a need for training on the appropriate techniques of results communication while adhering to core principles.

### Compliance with core principles of MDAs

Confidentiality of information, no name, no shame, and no blame are the principles of good practice of clinical audit required by the national and international reference tools [3, 4]. CE Armstrong and al. in Tanzania in 2014 pointed out the importance of these standards in their study in this study the majority of participants were in favour of adherence to these principles [17]. Therefore, these principles should be applied rigorously [3–5]; and as recommended in the study by Van Hamersveld et al. [15]. Complying with core principles would promote the safety of actors and frank discussions contributing to improving the quality of health care provision [3–5, 15]. The confidentiality of the information was overall adhered to in our study. Most of the stakeholders are healthcare workers ordinary entitled to the confidentiality of medical information. This may have facilitated the adherence to this principle which needs to be consolidated.

The other core principles of MDAs were less adhered compared to confidentiality. Indeed, no name, no shame, no blame principles varied from one HD to another. Anonymity was the least respected principle. Armstrong et al. also noted lack of “shame and blame” in their study in Tanzania [17]. Complying to core principles was found overall challenging in the real and peripheral context of the health district as it was in experimental studies conducted at the third level of the health system in Ouagadougou [16] in Benin [9], and in Nigeria [8], respectively in 2008, 2010, and 2013. The difficulties encountered in the natural situation of our study pertained to the inadequate skills of actors in the field of MDAs, resulting in non-compliance with the prescribed rules when implementing clinical audits. The small number of staff members involved in health care provision to child birthing women per facility, the relative few number of maternal deaths and shortcomings in the programming of MDAs were identified as factors that favoured the breach into the anonymity. Therefore, for better compliance with the core principles, there is a need for capacity building and awareness raising among actors through initial training, and refresh training including supervision (district staff by regional hospital staff, regional hospital staff by the University hospital staff), improving accessibility to audit tools and their use. Compliance with principles will facilitate practice of maternal death reviews in health facilities and even that of other types of clinical audits like audit of near miss and audit based on the criteria.

### Limitations of the study

Our study faced the challenges described by V.C. Thorsen et coll. in 2014 in Malawi [18] for any study investigating MDAs. Shortcomings in record keeping may have played to undermine the quality of the information. Some key aspects of the quality such as interpersonal communication would have been best appreciated through a direct observation of MDAs sessions. In the face of all these challenges our methodology has a number of strengths: we considered only the deaths that occurred during the year preceding our study to improve the availability of records and we triangulated information from different sources to cross validate the findings. The principle of saturation of information was used in data collection to ensure that all relevant aspects are covered. Our findings can reasonably be hold for representative of the conduct of MDAs in health districts in Burkina Faso.

## Conclusion

Our study showed that MDAs programming in health districts remain very often uneven if not completely absent and the quality of communication among stakeholders at the various stages suffer several shortcomings, failing very often to target the comprehensive relevant audience and to preserve the anonymity. While a high level of adherence to confidentiality was reported, there was lower adherence to other core principles in the conduct of MDAs. Identifying barriers to the compliance with these quality standards is crucial for improving the practice in a natural situation at the peripheral level of the health system.

## Abbreviations

CUS: Care unit supervisor
DTM: District management teams
HD: Health districts
MCSU: Medical Center with Surgical Unit
MDG: Millennium Development Goal
NHPHF: Nurse head of primary health facilities (NHPHF)
SDG: Sustainable Development Goals
TUS: Technical unit supervisor
